# Wharton jelly-derived mesenchymal stem cell exosomes induce apoptosis and suppress EMT signaling in cervical cancer cells as an effective drug carrier system of paclitaxel

**DOI:** 10.1371/journal.pone.0274607

**Published:** 2022-09-15

**Authors:** Burcin Irem Abas, Gulen Melike Demirbolat, Ozge Cevik

**Affiliations:** 1 Department of Medicinal Biochemistry, School of Medicine, Aydin Adnan Menderes University, Aydin, Turkey; 2 Department of Pharmaceutical Technology, Faculty of Pharmacy, Acibadem University, Istanbul, Turkey; Università degli Studi della Campania, ITALY

## Abstract

Mesenchymal stem cells can be obtained and multiplied from various sources and have a very high capacity to release exosomes. Exosomes are nano-sized extracellular vesicles containing biological signaling molecules. This study aimed to determine the effect of MSC-derived exosomes as a drug delivery system for paclitaxel in cervical cancer cells. In this study, human MSC were isolated from wharton jelly of umbilical cord tissue (WJ-MSC), and cells were characterized by CD44, CD90, CD105, and CD34 staining. Exosomes were released in WJ-MSC cells with serum-starved conditions for 48 hours, and particle sizes and structures were examined with zeta-sizer and TEM. In addition, exosomes CD9, CD63, and CD81 markers were checked by western blot. Paclitaxel was loaded into exosomes (Exo-PAC) by electroporation and then incubated with Hela cervical cancer cells for 24 hours. TGF-β, SMAD, Snail, Slug, β-catenin, Notch, Caspase-3, Caspase-9, Bax, Bcl-2 protein and gene expression levels were analyzed in Hela cells. As a result, low concentration Exo-PAC induced apoptosis, and suppressed epithelial-mesenchymal transition proteins in Hela cells. In this study, it has been demonstrated that WJ-MSCs can be used as drug delivery systems for cervical cancer if exosomes are produced scalably in the future.

## Introduction

Stem cells are unspecialized cells that have a remarkable ability to be constantly regenerated in the human body. Sources of stem cells include bone marrow, umbilical cord, cord blood, and adipose tissue [1]. Although the umbilical cord is traditionally seen as biological waste, it is a rich source of stem cells. Unlike stem cells from other sources, these umbilical cord tissues are readily available in large quantities, and the stem cells are quickly recovered without undergoing any invasive procedure [2]. Wharton’s jelly is a stem cell source consisting of connective tissue composed of mesodermal cells in the umbilical cord [3]. UC-derived MSC’s unique properties make them attractive alternatives in cell therapies and regenerative medicine [4]. Stem cells isolated from Wharton’s jelly show mesenchymal fibroblast-like morphology with the ability to self-renew and differentiate into neuronal, osteochondral, adipocytic, and muscle derivatives [5]. In addition, Wharton jelly yields stem cells that are positive for MSC markers (CD44, CD90, and CD105) but lack endothelial markers (CD144, CD146, and CD34). MSCs isolated from Wharton’s jelly respond rapidly to tissue damage and induce angiogenesis due to rapid adhesion [6]. Evidence is that they exert their effects through exosomes from extracellular vesicles (EVs) [7]. Exosomes are part of EVs of a specific size (40–160 nanometers), which have a double-layered membrane-enclosed lipid and can be found in body fluids such as blood, urine, saliva, and amniotic fluid [8]. The biological function of the exosome relies on its bioactive cargoes, such as lipids, metabolites, proteins, and nucleic acids, which can be delivered to target cells. With the development of research methodologies and techniques, people have realized that exosomes represent a new mode of intercellular communication and contribute to a wide variety of biological processes, including health and many diseases, including cancer [9]. Exosomes and their biomolecules can be serve as prognostic cancer markers, therapeutic targets, and even anti-cancer drug carriers. While exosomes are thought of as residues left from cells, they have played a role in both the diagnostic field and understanding transitions in molecular mechanisms in recent years. In particular, studies have focused on how they affect physiological and pathological processes with the molecules they carry after they are released from the cells. Because MSCSs have a high self-renewal capacity, they have high paracrine activity and secrete many exosomes [10]. MSC exosomes play a role in developing many diseases and are also used therapeutically. They not only participate in the process of tissue repair and injury [11, 12] and have specific therapeutic effects on cardiovascular diseases [13, 14] and neurological diseases [15], but also the liver. They can alleviate the damage and be used in treatment [16]. On the other hand, the issue of obtaining these exosomes from stem cells in new approaches regarding both their roles and areas of use leads to debates. For this reason, it is more critical to use tissues easily obtained by non-invasive methods rather than invasive procedures in stem cell isolation and use. The placenta and umbilical cord are mainly disposed of as biological waste after birth and not used for recycling [17, 18] The fact that the placenta and umbilical cord tissues are available in large quantities and are readily available is essential in stem cell isolation and the development of derivatives for use in therapy [2, 4].

This study investigated the potential of exosomes released from mesenchymal stem cells isolated from the umbilical cord as a carrier of anti-cancer drugs such as paclitaxel for cervical cancer cells.

## Materials and methods

The study design was approved by the ethics committee of Aydin Adnan Menderes University School of Medicine Research Ethics Committee (Protocol number 09012020/23). The study was conducted according to the criteria set by the Declaration of Helsinki, and each subject was informed in writing before participating in the study. Formal written informed consent was obtained from the participants before tissue collection. This study was carried out in Aydin Adnan Menderes University Hospital Gynecology Clinic. The umbilical cord was taken from the mother who gave birth to a healthy baby (>38 weeks) between 20-and 40 ages.

### Isolation and characterization of WJ-MSC cells from UC

UC tissue was placed in phosphate-buffered saline (PBS, 100 U/ml penicillin, 100 μg/ml streptomycin, 2 μg/ml amphotericin B), and Wharton jelly (WJ) was dissected. WJ was incubated with collagenase (1 mg/mL type I) and hyaluronidase (0.7 mg/ml) for 1 hour at 37°C and centrifuged at 340xg [19]. DMEM/F12 supplemented with 15% FBS was added to the cell pellet and incubated at 37°C with 5% CO_2_. Cells were followed for 21 days and replaced with fresh medium every four days. The morphology of WJ-MSC cells was checked under the microscope. CD44, CD90, CD105, and CD73 staining were detected by flow cytometric analysis in WJ-MSC cells at the 21^st^-day passage. The immunofluorescence method performed WJ-MSC cell surface receptors CD90, CD105, and CD44 staining [20].

### Isolation and characterization of exosomes from WJ-MSC

MSC cells were grown in 75 cm^2^ flasks in FBS-free (starved) DMEM/F12 medium for 48 hours. First, the medium was collected to obtain exosomes released from fasting cells over 48 hours. Afterward, the media were centrifuged for 10 minutes at 13,000xg and 10 minutes at 45,000xg to separate cells and large vesicles. It was then centrifuged at 110,000xg for 5 hours (Beckman Coulter) in an ultracentrifuge. Finally, the supernatant was discarded, and the pellet was suspended with PBS.

Characterizations of isolated exosomes were checked by western blotting for CD9, CD63, and CD81 markers. Exosome pellets were denatured 1:1 in 2X Sample loading buffer (4% SDS, 20% glycerol, 10% β-mercaptoethanol, 0.004% bromphenol blue and 0.125 M Tris HCl, pH 6.8) for 5 minutes at 95°C. SDS-PAGE gels were prepared, and samples containing five μg/mL protein were loaded. The gels were loaded with PVDF membrane, and blotting was performed at 25 Volt and 1 Amp for 30 minutes (Biorad Transblot Turbo). The membrane was blocked with 3% BSA, and after 2 hours, the primer was applied. Incubate with antibody overnight at +4° C. The membrane was washed three times with TBST (20 mM Tris, 154 mM NaCl, 0.1% Tween 20) Membrane was washed with secondary antibody (1:1000, HRP conjugated sc-2030, sc- 2020) was incubated for 2 hours in room temperature. A chemiluminescent substrate was placed on the membrane, and analysis was performed on the imaging system (Syngene G:Box). A zeta sizer analyzed the quantities, sizes, and charges of exosomes. SEM electron microscopy method was used to scan nano-sized structures after their measurements.

### Paclitaxel loading into WJ-MSC exosomes

An anti-cancer agent, paclitaxel, was loaded into the cell of exosomes [21, 22]. Exosomes (Exo) were suspended in electroporation buffer (1.15 mM potassium phosphate (pH 7.2), 25 mM potassium chloride and 21% (vol/vol) OptiPrep). This method creates small pores in the exosome membrane by applying an electric field to the exosomes suspended in a conductive solution. The electric current disrupts the phospholipid bilayer of exosomes, thus causing the formation of temporary pores. The drugs then diffuse into the interior of the exosomes via the pores [23]. Then one μg of paclitaxel (PAC) was mixed with the exosome suspension and transferred to sterile electroporation cuvettes. The cuvettes were loaded with paclitaxel by applying current at 160 V and 500 μF. Then, paclitaxel-loaded exosomes (Exo-PAC) were separated by centrifugation at 90.000 rpm for one hour. Paclitaxel loading into exosomes was checked with a UV-VIS spectrophotometer. In order to determine the PAC loading efficiency to exosomes, the PAC solution prepared as the stock was used, the spectrum was taken in the range of 200–700 nm, and the maximum peak value was 225 nm. At this wavelength, the values in the supernatant were measured before and after exosome loading. It was calculated indirectly by proportioning according to the amount of PAC with the known value. As a result, PAC loading efficiency was 62% in exosomes. PAC release experiments from Exo-PAC were performed in pH 7.4, 6.3, and 5.50 buffers in PBS on samples taken at 0–72 hour time intervals, and release amounts were determined. It was then used for cellular studies with the Exo-PAC.

### Cytotoxic effects of Exo-PAC effects in Hela cells

After obtaining exosomes and Exo-PAC, experiments were performed on human cervical cancer cell Hela and normal cells (L929 mouse fibroblast). Hela and L929 cells were used in a DMEM medium containing 10% FBS, Penicillin-Streptomycin (100 units), L-Glutamine, and NaHCO3. Cells were incubated at 37°C in an environment containing 5% CO2 and 95% humidity and multiplied. Then, cells were seeded into 96 well plates and incubated for 24 or 48 hours with Exo or Exo-PAC at concentrations of 0.1 μg, 1 μg, 10 μg, and 100 μg. IC50 concentrations on the cytotoxic effects of exosomes were determined by the MTT (3-[4,5-Dimethylthiazole-2-yl]-2,5-diphenyl tetrazolium bromide) test.

### Measurement of Hela uptake of Exo-PAC

1x10^4^ Hela cells were seeded in 4 well chamber slides, and Exo or Exo-PAC was added to the medium at 10 μg concentrations and incubated for 24 hours. DiO (green) and DAPI staining were performed and images were taken under a fluorescent microscope and the status of exosomes in the cell was determined. 1x10^4^ Hela cells were seeded into 24 well plates and incubated with 10 μg of Exo or Exo-PAC for 24 hours. After incubation, the amount of paclitaxel in both the medium and cell pellets [24] was measured with a fluorometer.

### Annexin-V binding of Exo-PAC in Hela cells

1x10^5^ Hela cells were seeded into six-well plates and incubated with 10 μg of Exo or Exo-PAC for 24 hours. Exo or Exo-PAC (10 μg) was added and incubated for 24 hours. The cells were removed by adding 1 mL of trypsin-EDTA after incubation, and the supernatant was discarded at the end of centrifugation at 800xg. The pellet was resuspended in 1 mL of PBS containing 1% FBS, and 100 μL of Annexin V Cell Reagent (Muse Annexin V kit) was added to the cells and incubated in the dark for 20 minutes. At the end of incubation, it was analyzed in the Muse Cell Analysis System (Millipore, Germany).

### Cell colony formation and Scratch assay

Hela cancer cells were seeded as 500 cells/well in 6 well plates [25]. It was incubated with 10 μg of Exo or Exo-PAC for 15 days. Afterward, each well was fixed with methanol+acetic acid (3:1) for 5 minutes and stained with 0.5% Crystal violet dye for 20 minutes. First, the size of the colonies was checked under the microscope, and their morphological images were examined. Then, 2 mL of methanol was added to the colonies, and the amount of absorbed crystal violet was measured spectrophotometrically at 570 nm. Finally, the results were calculated by comparing the colony-forming ability.

A Scratch test was performed to measure the migration potential of tumor cells [26]. Cells were seeded into 12 well plates and scratched with a 100μl yellow pipette tip. Exo and Exo-PAC were incubated for 24 hours, and cell images were taken under an inverted microscope. The gap in the 0h and 24h images was calculated, and the viable adherent cells were counted.

### Protein and gene expressions in Hela cells

Hela cells were seeded in 6 well plates and incubated with Exo (10μg) and Exo-PAC (10μg) for 24 hours. After incubation, cells were washed two times with PBS. TGF-beta, SMAD, Snail, Slug, B-catenin, Notch, Caspase-3, Caspase-9, Bax, Bcl-2 markers were used by western blot [27]. For gene expression studies, cells were lysed with RNA lysis buffer. Total RNA isolation (Invitrogen, AM1912) and cDNA synthesis (Applied Biosystems 4368814) were performed using a commercial kit. Primers specific for TGF-beta, SMAD, Snail, Slug, B-catenin, Notch, Caspase-3, Caspase-9, Bax, Bcl-2 (Sigma KiCqStart Primers), and SYBR Green Master Mix (Applied Biosystems, A25742) were used. After preparing the mixture with a total reaction of 20 μl in each well (SYBR Green Master Mix: 10 μl +F primer: 1 μl+ R primer: 1 μl + cDNA), 95°C for 5 min, 95°C 30 sec, 55°C 30 qRT-PCR was set up (ABI, StepONE Plus) as 40 cycles at 72°C 30 sec, 72°C 4 min. GAPDH was selected as the housekeeping gene, and the 2^–ΔΔCt^ method was used for calculations.

### Statistical analysis

All experiments were carried out with at least three replications. Statistical analyzes were performed using GraphPad Prism 7.0. Comparisons between groups in the analyzes were made using the independent t-test, and the significance levels were accepted as values below 0.05.

## Results

### Umbilical cord stem cell isolation and WJ-MSC characterizations

**Fig 1:**
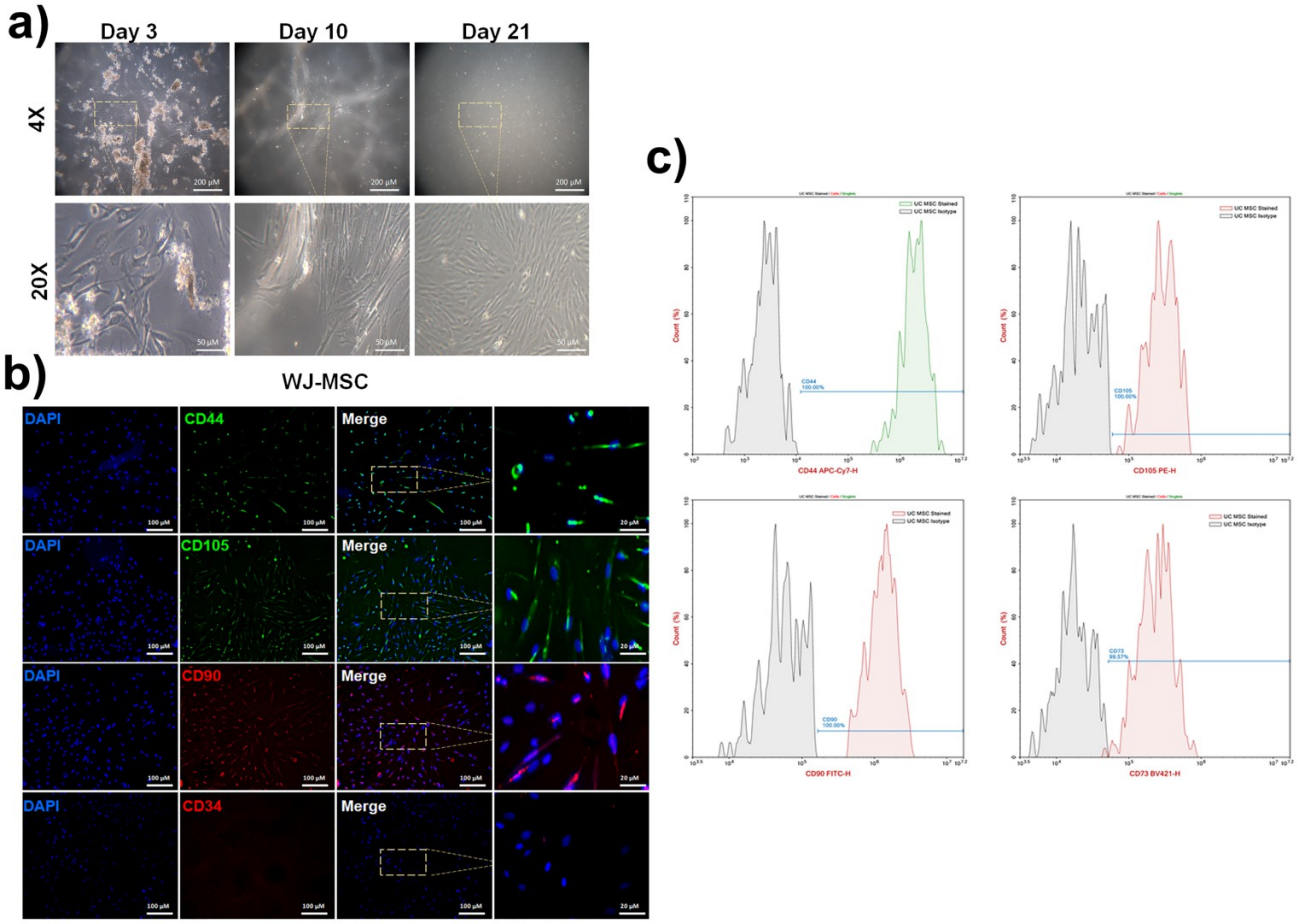
Morphology and characterization of WJ-MSC cells a) Morphological images of WJ-MSC cells on day 3, day 10, and day 21 with scale bar 200 μm and 50 μm respectively b) Binding of WJ-MSC CD44, CD90, CD73, and CD105 by flow cytometry c) WJ-MSC also CD34, CD44, CD90, and CD105 staining by IF staining.

Morphological images of WJ-MSC cells on day 3, day 10, and day 21 are shown in Fig 1a. The proliferation capacity of WJ-MSC cells increases depending on the day, and their morphological features resemble branched active fibroblasts. It was determined that WJ-MSC cells preserved their morphology for up to 21 days and had fusiform (spindle) morphology. In determining the phenotypes of WJ-MSC cells by flow cytometry, the binding of CD44, CD90, CD73, and CD105, which are the primary markers of MSC, was found to be 99.8% and above (Fig 1b). Cells isolated from WJ-MSC had positive CD44, CD90, and CD105 staining in immunofluorescent staining, while CD34 staining was negative (Fig 1c).

### WJ-MSC exosomes purification, characterization and SEM analysis

**Fig 2:**
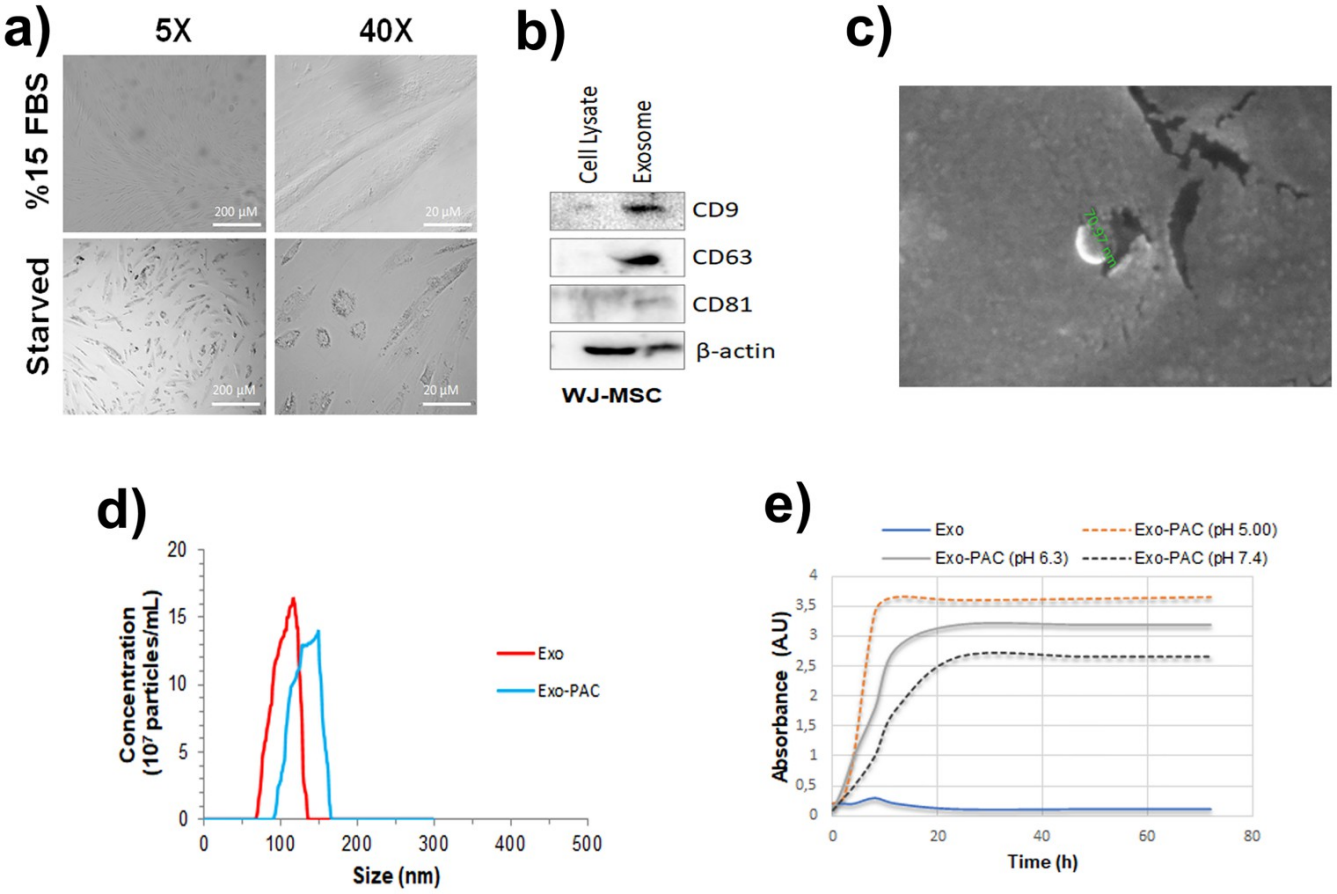
WJ-MSC exosomes characterization a) Morphologies of WJ-MSC cells grown in serum and serum-free medium b) CD9, CD63, and CD81 western blot bands of exosomes isolated from WJ-MSC c) Exo and Exo-PAC SEM analysis images d) Exo and Exo-PAC size analysis e) Exo and Exo-PAC time-dependent PAC release graph.

Exosome release from WJ-MSC cells was performed from the medium by starving the cells in serum-free media (starved) for 48 hours. Images of cells growing in serum and serum-free medium are shown in Fig 2. When the images of the cells are examined, it is seen that the cytoplasmic connections between each other decrease in the cells grown in a serum-free medium, the cells become more sparse and exhibit apoptotic morphology, the release of apoptotic bodies increases, and the fibroblast appearance of the cells disappear. MSCs with high resistance and adhesion tendencies began to secrete exosomes after 48 hours of fasting, as the cells increased exosome release under stress or starvation conditions (Fig 2a). WJ-MSC exosomes were detected by western blot to carry cell surface markers CD9, CD63, and CD81. CD9 and CD63 were dominant in exosomes, while CD81 was found in lower amounts (Fig 2b).

SEM imaging was performed to measure the shape and size of exosomes. After the analysis, it is seen that the Exo isolated from WJ-MSC cells is 70.97 nm in size, and after PAC loading, the Exo-PAC is 128 nm in size and retains its spherical shape (Fig 2c). Zeta-sizer was used in studies on whether there was a change in size and load after PAC loading into exosomes. It was observed that the size of Exo-PAC (82±32 nm) was slightly larger than that of Exo (118±21 nm), and the size changes were at a level that did not prevent entry into the cell (Fig 2d). The release graph in buffers at different pHs depending on the time of PAC loaded into exosomes is shown in Fig 2e. It was observed that PAC release from Exo-PAC was higher in a short time (8 hours) in pH 5.00 buffer, whereas this release decreased in pH 7.4 buffer and appeared later (24 hours).

### Effect of Exo and Exo-PAC in Hela cells

**Fig 3:**
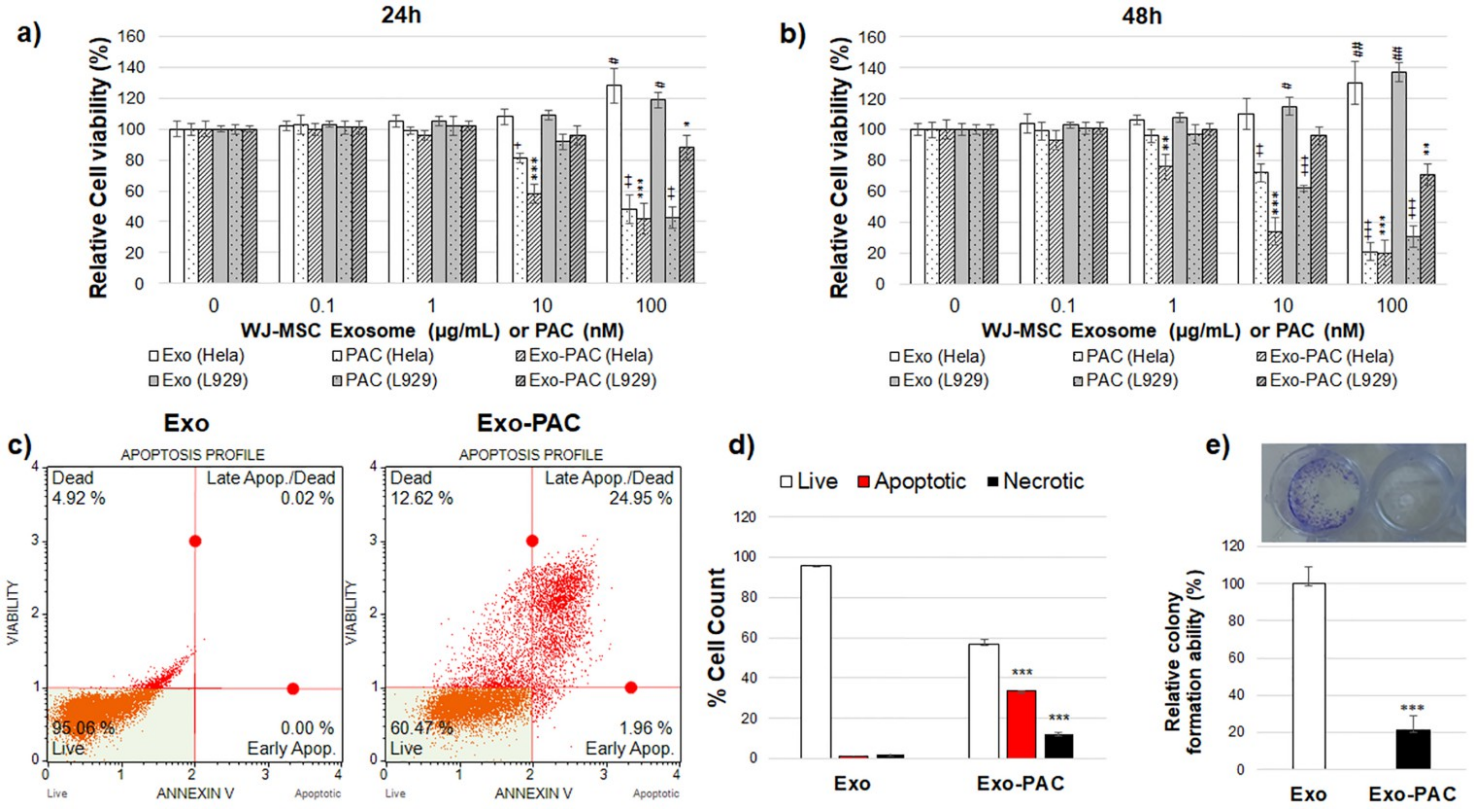
Exo and Exo-PAC application to Hela and L929 cells a) 24 hours and b) 48 hours cell viability graph c) Annexin V binding graphs of Exo-PAC in Hela cells d) Annexin V binding levels in Hela cells e) Colony formation levels in Hela cells (**p<0.01, ***p<0.001 compare to Exo).

In MTT studies, the PAC IC50 value was found at a concentration of 18.62±2.33 nM in Hela cells and 67.42±5.81 nM in L929 cells. The IC50 value in Exo-PAC was calculated as 13.21±1.82 μg/mL in Hela cells. The IC50 value could not be calculated because cell viability was high in Exo applied cells. MTT experiments using the L929 healthy cell line determined that Exo application alone increased cell proliferation, and the IC50 value could not be calculated. In L929 cells, the IC50 value was found to be 197±4.65 μg/mL in Exo-PAC application (Fig 3a and 3b).

Exo alone increased cell viability at a 100 μg/mL concentration both in 24 and 48 hours in Hela and L929 cells (p˂0.05, p˂0.01) and did not change at other concentrations. PAC treatment at 10 nM and 100 nM concentrations decreased cell viability in Hela and L929 cells for 24h (Fig 3a). Exo-PAC treatment showed effects at 10 μg/mL and 100 μg/mL concentrations in Hela cells (p˂0.001), while it reduced cell viability only at 100 μg/mL concentration in L929 cells for 24h (p˂0.01). PAC treatment decreased cell viability at 10 and 100 nM concentrations in both Hela and L929 cells at 48 hours. Exo-PAC between 1 and 100 μg/mL significantly decreased cell viability in the Hela cells in 48 hours (p˂0.01, p˂0.001). In L929 cells, on the other hand, it decreased cell viability by acting at a concentration of 100 μg/mL (p˂0.01, Fig 3b). Paclitaxel loaded into these exosomes entered the cell and accumulated, allowing it to show the same effect as paclitaxel administered alone.

Annexin-V binding levels are seen in Fig 3c when ten μg/mL Exo or Exo-PAC are applied. The number of viable cells in the Exo group was 96.03±2.34% in Hela cells and 57.03±2.34% in cells treated with Exo-PAC (p<0.001). The number of apoptotic cells (early and late apoptosis) in the Exo group was 0.81±0.05%, and 33.71±1.55% in the cells treated with Exo-PAC (p<0.001). Necrotic cell counts were determined as 2.11±1.02% in Exo group cells, and 12.03±2.03% in Exo-PAC treated cells (Fig 3d, p<0.001).

In order to evaluate the metastatic effect in colony formation, cells are evaluated in terms of their capacity to proliferate alone in their region and how they behave. The colony formation potential of Exo and Exo-PAC for 14 days is shown in Fig 3. In addition, statistically significant inhibition of colony formation was determined by Exo-PAC application (Fig 3e, p<0.001). A Scratch test was performed to measure the migration potential of Exo and Exo-PAC in tumor cells. In the scratch experiment, it was seen that Exo-PAC did not change the gap closure. However, since the PAC in Exo-PAC is a microtubule inhibitor, it suppresses the migration abilities of cells by blocking their division, adhesion, and movement. In addition, since Exo-PAC inhibits the adhesion of cells by suppressing the adhesion tendency of cells, it is not sufficient to evaluate them only in terms of gap closure. However, the number of cells should also be controlled. It has been shown that Exo-PAC significantly reduces both gap closure and the number of adherent cells (S1 Fig, p<0.001).

### Exo and Exo-PAC protein expression changes in Hela cells

**Fig 4:**
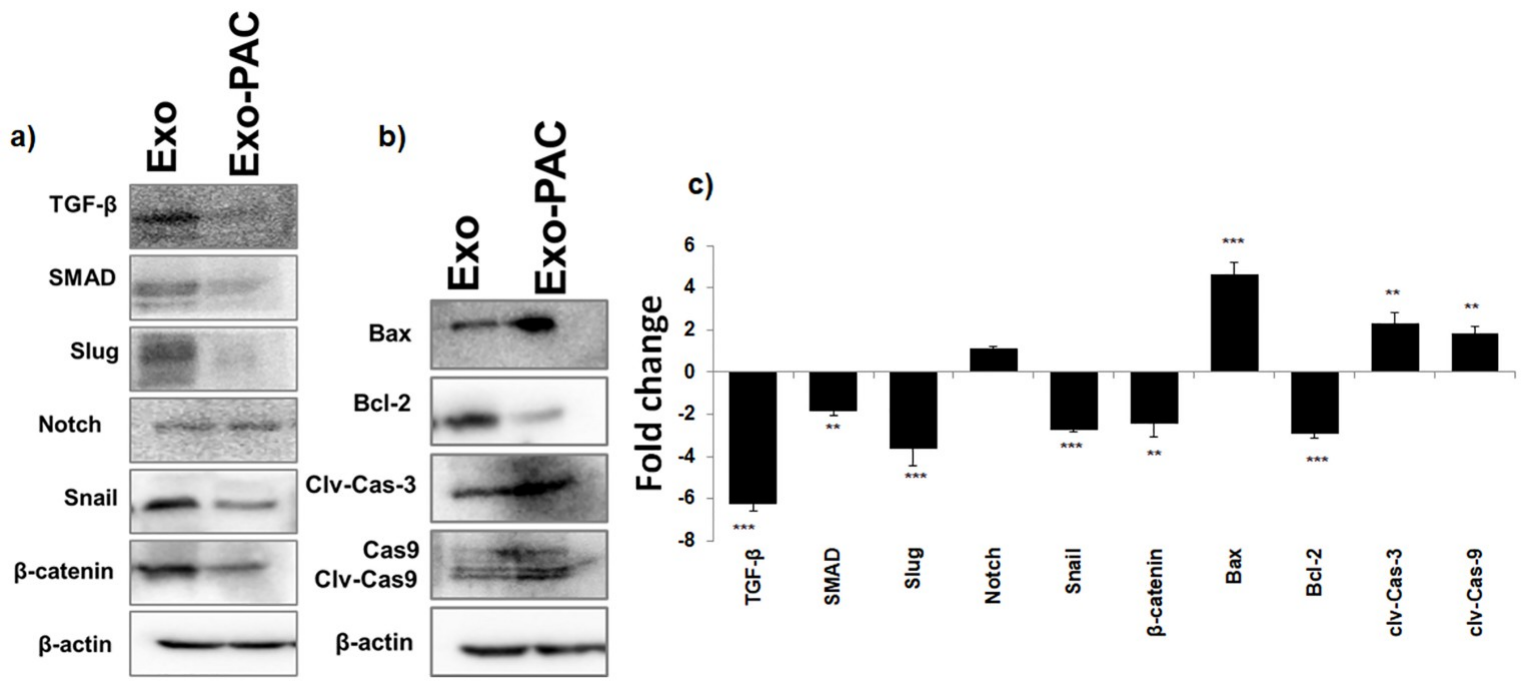
Western blotting protein expression changes a) Band image of EMT-related proteins, b) Band image of apoptosis-related proteins, c) Fold change graph.

Epithelial-Mesenchymal Transition (EMT) activation and expression differences of proteins that play an essential role in apoptosis were detected by western blot. The band changes of protein expressions in Exo and Exo-PAC application in Hela cells are seen in Fig 4.

When the protein expression changes associated with epithelial-mesenchymal transition were analyzed (Fig 4a), the TGF-β level decreased 6.25±0.35 (p˂0.001) times in the Exo-PAC group, SMAD level was 1.86±0.2 (p˂0.01) times, Slug level decreased 3.65±0.8 times (p˂0.001), Notch level changed 1.12±0.15 times. However, it did not increase significantly, and the Snail level was 2.75±0.1. In addition, it is observed that the β-catenin level decreased 2.46±0.6 times (p˂0.01) compared with the Exo group.

When apoptosis-related protein expression changes were examined (Fig 4b), the Bax level increased 4.62±0.6 (p˂0.001) times, Bcl-2 level decreased 2.95±0.2 (p˂0.001) times in the Exo-PAC group. The clv-Cas-3 level increased 2.3±0.5 (p˂0.01) times, and the clv-Cas-9 level increased 1.87±0.3 (p˂0.01) times compared to the Exo group. (Fig 4c).

### EMT and apoptosis-associated gene expression changes

**Fig 5:**
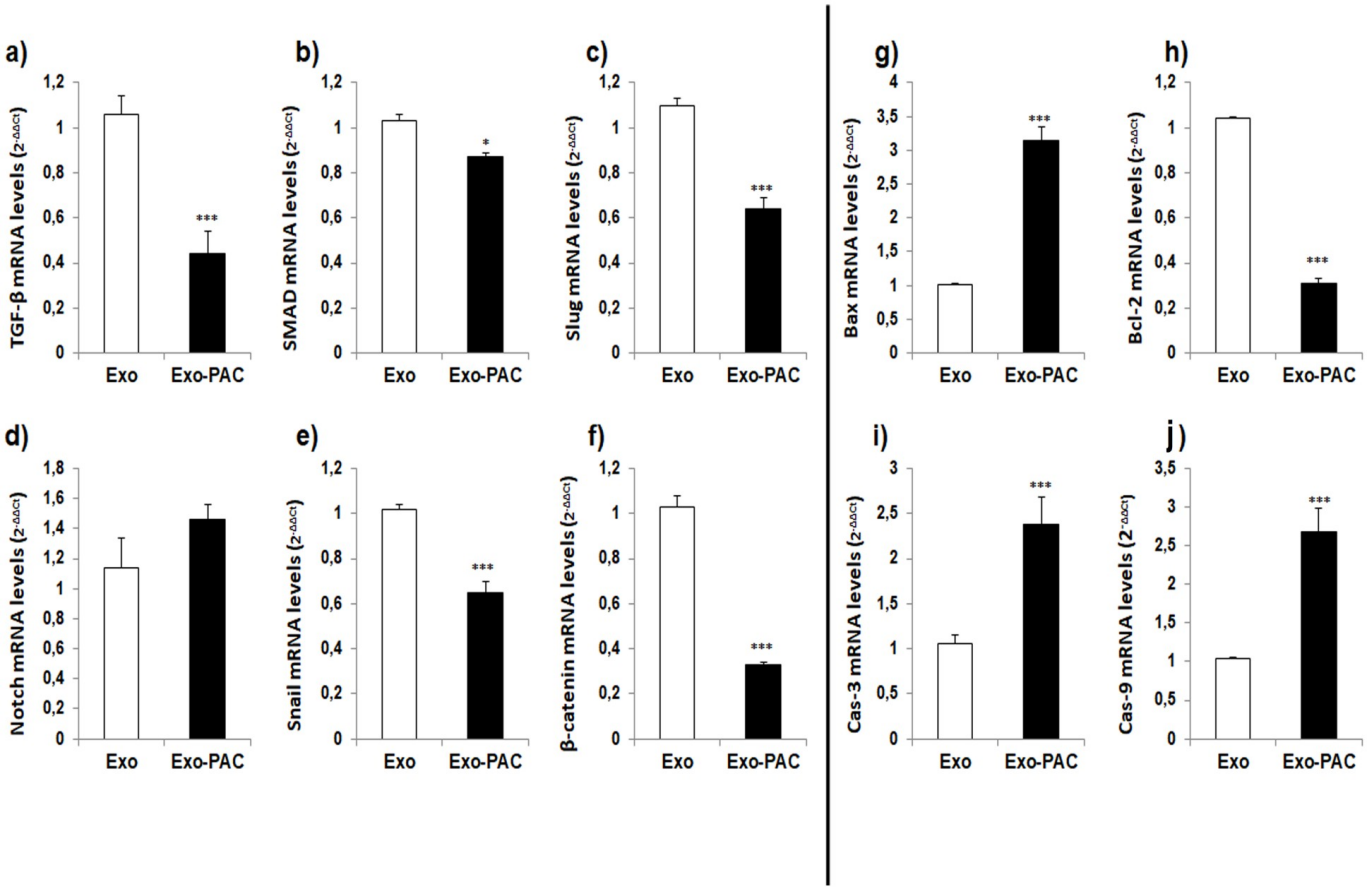
Graphs of mRNA levels of EMT and apoptosis-related genes.

The gene expression levels of TGF-β, SMAD, Snail, Slug, Notch, β-catenin, and apoptosis-associated Bax, Bcl-2, Cas-3, and Cas-9 genes related to epithelial-mesenchymal transition were analyzed by qPCR (Fig 5).

When gene expression changes associated with epithelial-mesenchymal transition were examined, TGF-β mRNA level was significantly decreased by 0.44±0.10 in the Exo-PAC group (p˂0.001), SMAD mRNA level was 0.87±0.02 significantly compared to the Exo group. In addition, there was a decrease (p˂0.05), Slug mRNA level was 0.64±0.05 significant decrease (p˂0.001), Notch mRNA level was 1.46±0.20 increase was not significant, Snail mRNA level was 0.65±0.02. It was observed that there was a significant decrease of 0.05 (p˂0.001) and a significant decrease of β-catenin mRNA level of 0.33±0.01 (p˂0.001) (Fig 5a–5f). When the gene expression changes associated with apoptosis were examined, the Bax mRNA level increased by 3.14±0.20 significantly (p˂0.001) and the Bcl-2 mRNA level decreased by 0.31±0.02 significantly compared to the Exo group (p˂0.001), Cas-3 mRNA level increased 2.38±0.31 significantly (p˂0.001), and Cas-9 mRNA level increased 2.68±0.20 significantly (p˂0.001) (Fig 5g–5j).

## Discussion

Cervical cancer is among the most common female malignancies globally and is detected at an early stage by human papillomavirus (HPV) screening. HPV is the leading cause of cervical cancer and can be transmitted sexually [28]. Cervical cancer progression depends on HPV and multiple heterotypic cell interactions that make up the tumor environment. In examining these effects, the potential of using exosomes, which play a role in cellular communication, as a diagnostic and therapeutic biomarker, is being investigated [29, 30]. Our study isolated mesenchymal stem cells from umbilical cord tissue wharton jelly and purified the exosomes they secreted out of the cell in vitro. We loaded paclitaxel, a taxane group drug, into these exosomes, turned them into functional exosomes, and evaluated their effects on cervical cancer cells.

A current challenge in exosome research is the lack of characterization of existing methodologies that assess exosomes’ availability, purity, and isolation from their conditioned media in cell culture and yield from complex biological fluids such as serum/plasma. For these reasons, exosome isolation is essential, and differential centrifugation consisting of ultracentrifuge is used as the "gold standard" for purification [31]. An alternative to ultracentrifugation is to concentrate from their conditioned media in large volume cell culture using ultrafiltration devices. In exosome separation, the search for alternative methods is still ongoing to ensure that the sample is less or more and the source of the sample is used correctly. Studies have shown that exosomes made with SEC (size exclusion chromatography) after ultracentrifugation is more pure isolation [32]. Our study found that the size of exosomes isolated from UC-MSC cells after ultracentrifugation separation was 70.97 nm, and this size increased slightly after PAC loading to 114±32 nm. Depending on the size of the isolated exosomes, the molecules they carry in their structures may differ. It is also known that the structures of exosomes isolated from different sources are different, even though they have the same size [33, 34]

Studies on drug loading into exosomes have shown that paclitaxel containing exosomes have a significant inhibitory effect on the in vitro proliferation of the human pancreatic cancer cell line CFPAC-1 and have the potential to act as drug carriers [35]. In another study, macrophage-derived exosomes containing paclitaxel have been shown to have a significant anti-tumor effect in the Lewis lung cancer metastasis model [36, 37]. However, there are also some differences in yield, content, function, and drug loading in exosomes from different sources. These can produce different therapeutic effects. For example, in a study by Kanchanapally et al. (2019), doxorubicin was successfully loaded into pancreatic stellate cells (PSCs) and pancreatic cancer cells (PCCs), and macrophage-derived exosomes [38]. In contrast, exosomes derived from PSCs have been shown to have the highest efficiency and high drug loading rate, while macrophage-derived exosomes have been shown to have the most potent anti-tumor activity. This effect indicates the specificity of exosomes from different sources. In a previous study, we used exosomes as a different carrier by loading the exosomes we isolated from Hela cells with docetaxel. We showed that re-administration of modified exosomes to the same Hela cells induces cell death [39]. In a study of exosomes developing biomimetic nanoparticles without losing the integrity of proteins, they first developed doxorubicin (DOX) loaded silicon nanoparticles. Then, DOX-loaded silicon nanoparticles were inserted into exosomes obtained from human hepatocarcinoma Bel7402 cells by passive loading. It has been shown that DOX-loaded silicon nanoparticles transported by exosomes have higher cellular uptake and cytotoxicity in total heterogeneity cancer cells and cancer stem cells. These results showed that it is a promising drug carrier for cancer chemotherapy and can be used to target exosome-biomimetic nanoparticles to tumor cells [40]. It was found that docetaxel loading (EXO-DTX) to exosomes isolated from A549 lung cancer cells increased the cytotoxic effect and significantly suppressed A549 cell proliferation. In addition, it has been determined that EXO-DTX triggers apoptosis, induces cell cycle arrest in the G2/M phase, and exerts an anti-cancer effect. In the in vivo experiment, it has been shown that EXO-DTX has a higher drug potential than free DTX and can be used as a good carrier system due to its faster entry into cells [41]. It is even suggested that the problems of water solubility and photosensitivity can be solved by these transport systems by loading photosensitizer anti-cancer drugs into exosomes [42, 43].

When the drug loading studies on exosomes isolated from mesenchymal stem cells were examined, it was found that paclitaxel was loaded from taxol group drugs. When the studies [44, 45] were examined, 10 μM paclitaxel was added to MSC544 MSC cells, and the cells were left to incubate for 24 hours. Cell media were collected, and exosomes were isolated. It was found that isolated paclitaxel decreased cell viability incubated with passively loaded exosomes A549 lung cancer cell, SKOV-3 ovarian cancer cell, and MDA-hybrid breast cancer cells. At the same time, it was shown that mammary tumors developed in NOD/SCID mice using MDA-hybrid breast cancer cells were reduced by intravenous injection of paclitaxel-loaded exosomes. Studies have shown that exosomes from MSC cells, effective on tumors by passive loading, can be used in different areas. In this study, it has been shown that paclitaxel is effective on Hela cells by active loading of exosomes (with electroporation), and it blocks migration by leading the cells to apoptosis with a higher effect at a lower concentration.

The use of paclitaxel, one of the chemotherapeutic drugs, as monotherapy or in combination with other therapeutic agents is a common strategy in treating cervical cancer. However, the response rate to treatment (percentage of patients with complete or partial response) ranges from 29% to 63%, mainly due to the achievement of chemoresistance [46]. Numerous studies have proven that the development of chemoresistance may be associated with the emergence of the EMT process [47, 48]. For example, EMT and chemoresistance have been reported in many cancer types in paclitaxel-resistant ovarian cancer cells [49], and gefitinib-resistant lung cancer cells [50] and cisplatin-resistant MDA-MB-231 cells [51]. For this reason, studies are continuing to elucidate the mechanisms of molecules that play a role in EMT and increase the efficacy of chemotherapeutic agents.

Paclitaxel has a high effect on cervical cancer cells Hela at the nanomolar concentration [52]. However, even if paclitaxel is placed in Hela cells above the inhibitory concentration (IC50: 112.53 μg/ml), it has been shown to develop drug resistance after a certain period [53, 54]. Therefore, paclitaxel should be transported with a more effective delivery system at a lower dose. Furthermore, the development of paclitaxel resistance during cervical cancer treatment has been positively associated with the epithelial-mesenchymal transition (EMT) process [55]. By EMT, epithelial cells remove their polarity and cell-cell adhesion properties and become mesenchymal stem cells by gaining migratory and invasive properties [56]. Thus, EMT promotes malignant progression and chemoresistance of cells. In a study, paclitaxel-resistant Hela cells were developed when paclitaxel was incubated in Hela cells at an increasing concentration (5–200 nM) for eighty days. It has been reported that EMT-related proteins (E-cadherin, N-cadherin, vimentin (VIM), fibronectin (FN), zinc finger E-box binding homeobox 1 (Zeb1), Snail, Slug) are increased in these resistant cells.

Furthermore, they showed that if inhibition of Notch signaling is achieved, it can partially restore paclitaxel sensitivity by reversing EMT in paclitaxel-resistant cervical cancer cells [57]. Therefore, preventing or delaying the emergence of paclitaxel resistance during cervical cancer treatment is a significant challenge in this field. In order to overcome this difficulty, it is necessary to fully understand the molecular mechanisms of chemoresistance and develop formulations or new carrier systems that can act at lower doses to facilitate the entry of the agents into the cell. In this study, it has been shown that when paclitaxel is loaded into WJ-MSC exosomes, it can affect Hela cells at lower concentrations, accelerate cell death, and inhibit chemoresistance by acting on EMT-related proteins.

Although there are many studies on cancer globally, an effective treatment strategy has not been determined yet. Using stem cells in cancer treatment is generally avoided because cancer cells have forms derived from stem cells. It is also predicted that stem cells can multiply uncontrollably with the growth factors they secrete. However, MSCs are thought to have antitumor effects due to their immune modulation capacity and ability to accumulate at the tumor site. Furthermore, MSCs are the only human cell type known to have a scalable capacity in the mass production of exosomes for drug delivery. MSC-derived exosomes are more efficient in transporting drugs than synthetic nanoparticles, as they exhibit biocompatibility/stability properties and a superior capacity for loading with various cargoes. In this study, it has been shown that the umbilical cord, which is considered a waste, is effective in transporting paclitaxel to target Hela cells at a lower dose by modifying mesenchymal stem cell exosomes. UC-MSC-derived exosomes are a potential candidate for both personalized therapy and the development of a scalable drug delivery system in the future.

## Supporting information

S1 Fig Hela cells motility was analyzed by Scratch assay of Exo and Exo-PAC application for 24h a) Representative images of the scratch assay and b) Quantification via the wound-healing area in 24h c) The number of adhering cells outside the gap (***p<0.001 compare to Exo).
(TIF)

S1 Raw images (RAR)

S2 Raw images (PDF)
