# Supplementary figures and images for: Wharton jelly-derived mesenchymal stem cell exosomes induce apoptosis and suppress EMT signaling in cervical cancer cells as an effective drug carrier system of paclitaxel
Source: PLoS One. 2022 Sep 15;17(9):e0274607. doi: 10.1371/journal.pone.0274607 (PMC9477505; doi:10.1371/journal.pone.0274607)

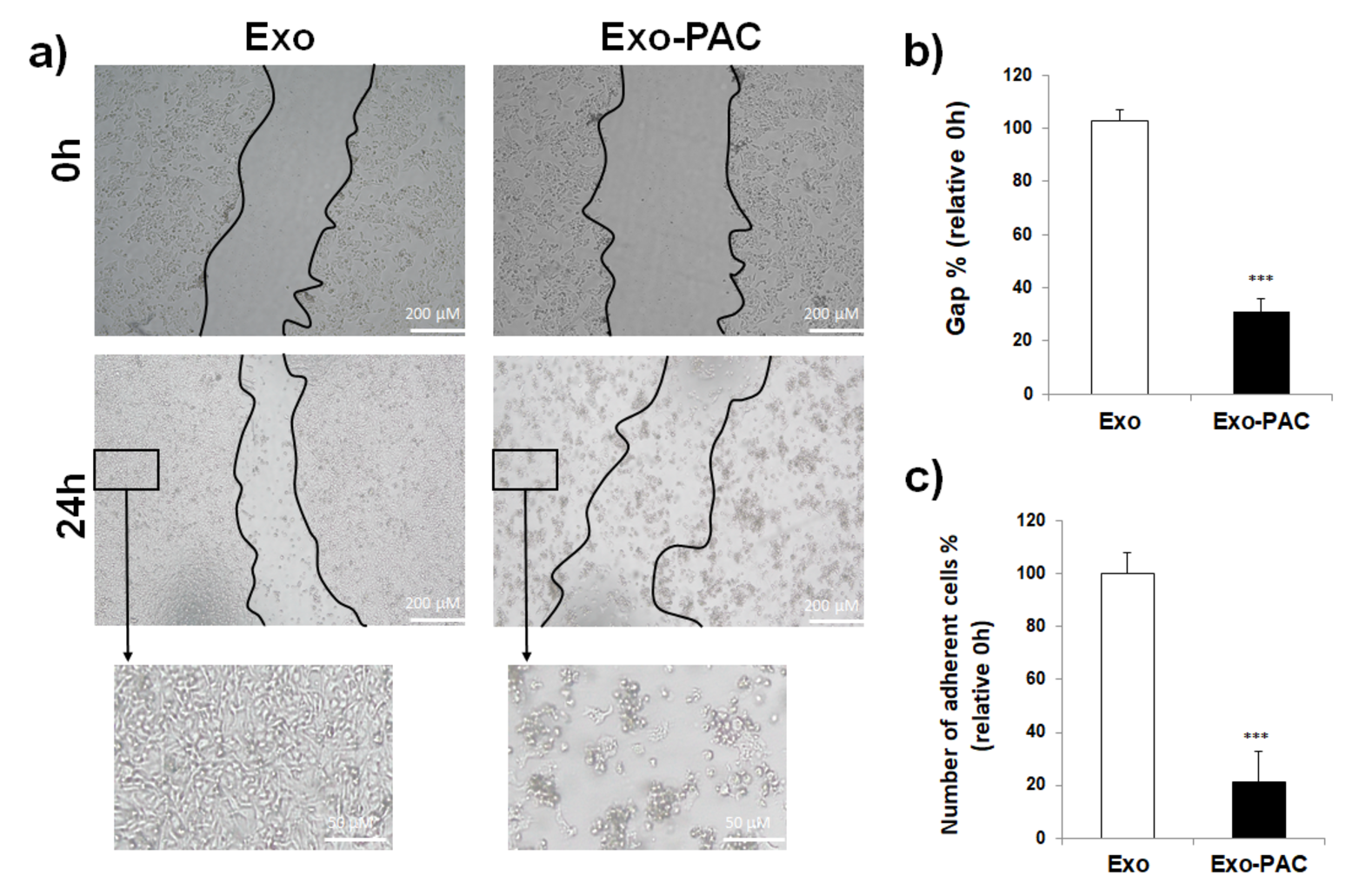

Supplement: S1 Fig — Hela cells motility was analyzed by Scratch assay of Exo and Exo-PAC application for 24h a) Representative images of the scratch assay and b) Quantification via the wound-healing area in 24h c) The number of adhering cells outside the gap (***p<0.001 compare to Exo). (TIF) [file pone.0274607.s001.tif]
