# Supplementary material for: Wharton jelly-derived mesenchymal stem cell exosomes induce apoptosis and suppress EMT signaling in cervical cancer cells as an effective drug carrier system of paclitaxel
Source: PLoS One. 2022 Sep 15;17(9):e0274607. doi: 10.1371/journal.pone.0274607 (PMC9477505; doi:10.1371/journal.pone.0274607)
Supplement: S2 Raw images — (PDF) [file pone.0274607.s003.pdf]

The bands used in the manuscript are framed in orange.

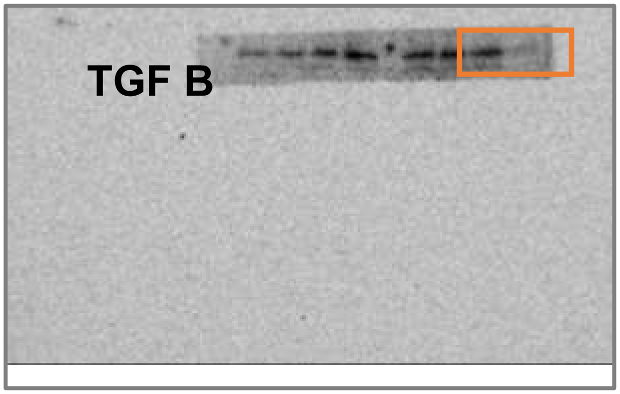

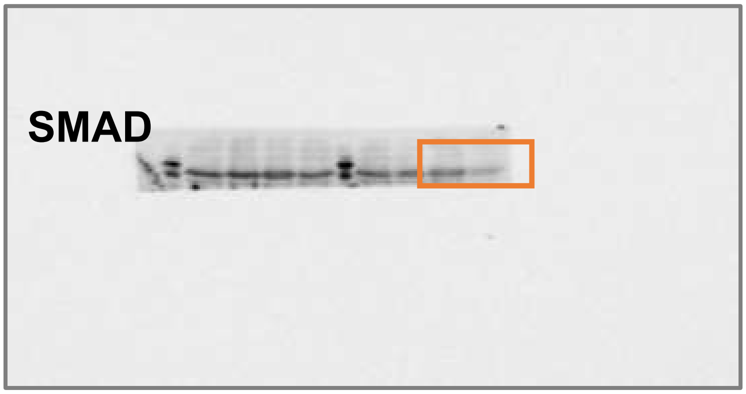

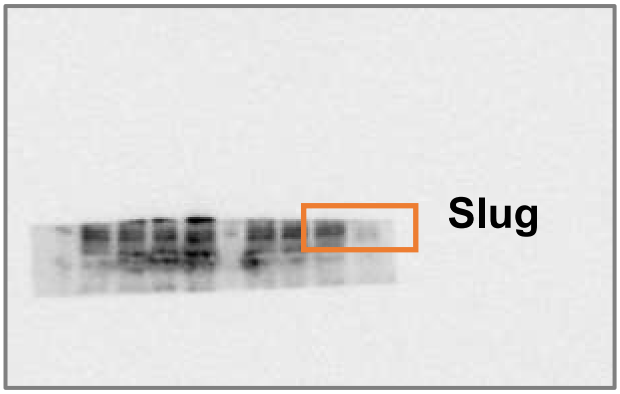

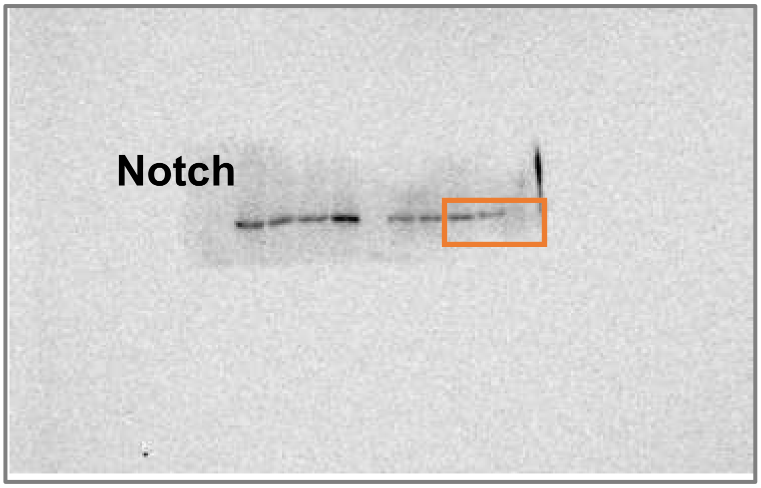

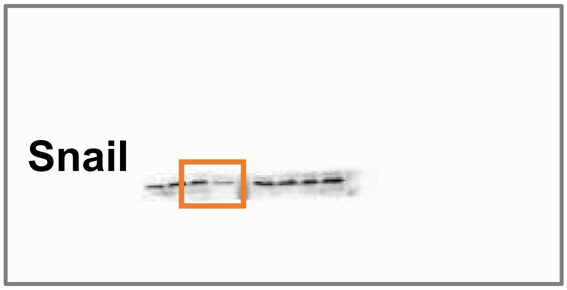
Snail

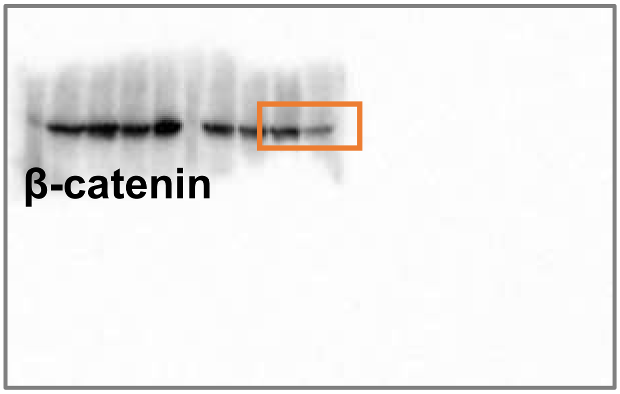
β-catenin

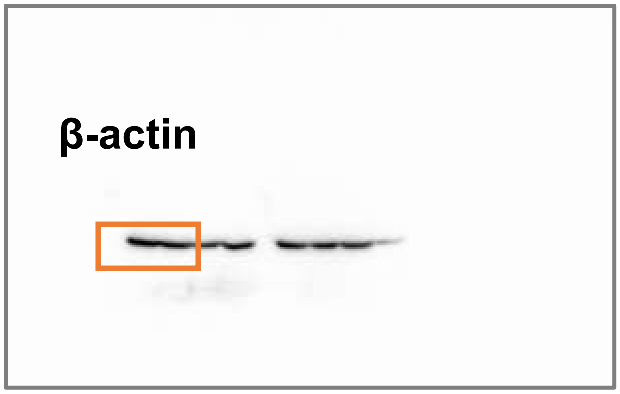
β-actin

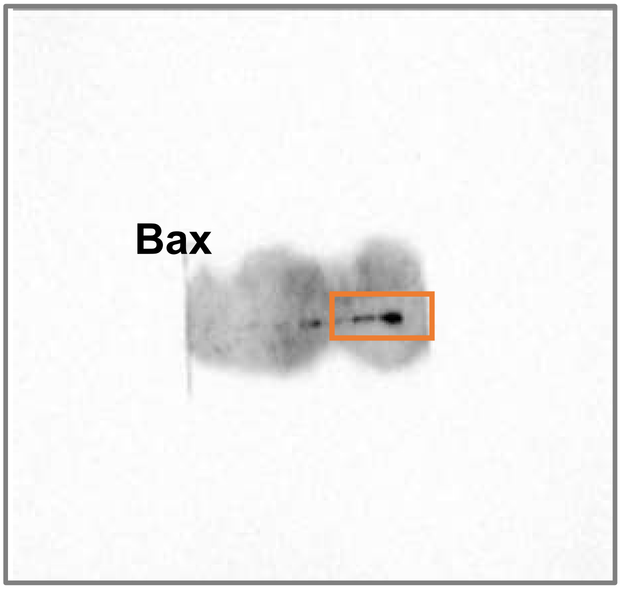
Bax

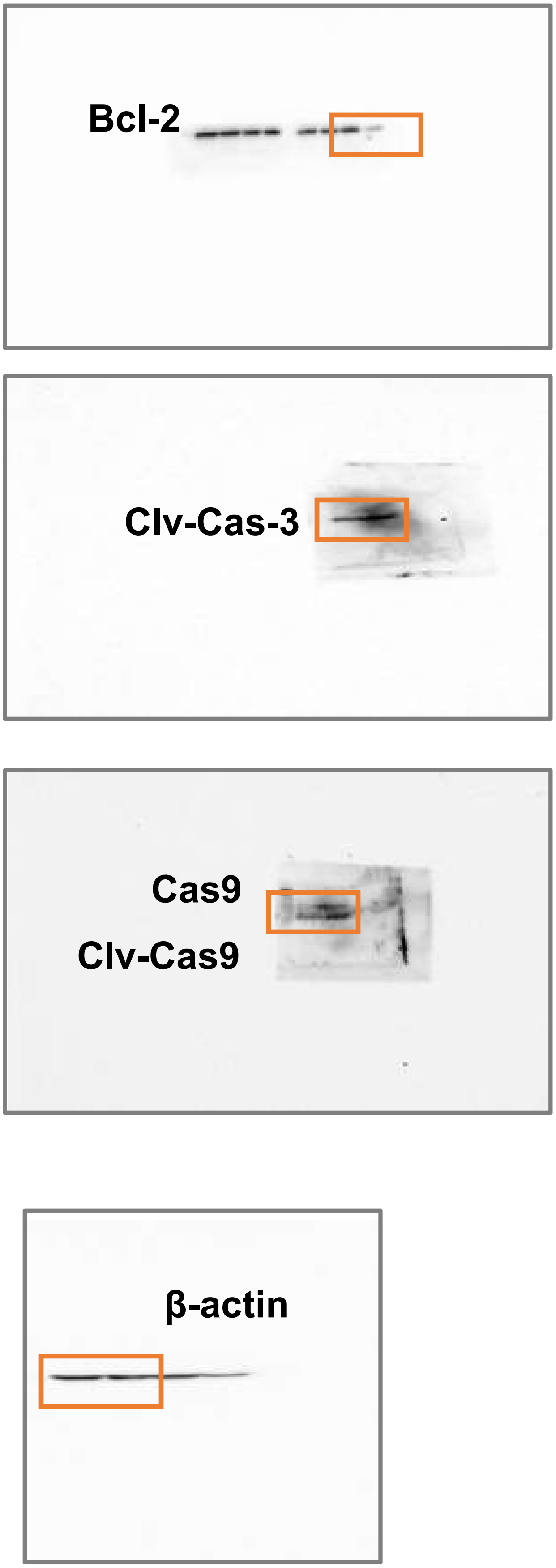
Bcl-2
Clv-Cas-3
Cas9
Clv-Cas9
β-actin
